# Individual and Contextual Socioeconomic Position on Type 2 Diabetes Risk in LMICs: A Multilevel Analysis

**DOI:** 10.1111/nyas.70400

**Published:** 2026-09-11

**Authors:** Reza Fahimi, Abdulaziz Alrubayyi, Muntaha Hassan, Thomas M. Barber, Olalekan A. Uthman

**Affiliations:** ^1^ Warwick Medical School University of Warwick Coventry UK

**Keywords:** low‐ and middle‐income countries, multilevel analyses, residence characteristic, socioeconomic status, type 2 diabetes mellitus

## Abstract

Type 2 diabetes mellitus (T2DM) represents a growing public health challenge in low‐ and middle‐income countries (LMICs), where urbanization and socioeconomic disparities amplify its burden. This study examined the influence of individual‐ and neighborhood‐level socioeconomic factors on T2DM risk using a multilevel framework. Cross‐sectional data from 14 nationally representative Demographic and Health Surveys conducted between 2013 and 2023 were analyzed, including 1,090,328 individuals within 38,174 neighborhoods. Multilevel logistic regression models were fitted to estimate associations between socioeconomic indicators and T2DM risk. Contextual variation was quantified using variance partition coefficients and median odds ratios. Higher individual‐level wealth was associated with increased odds of T2DM (OR = 1.30, 95% CI: 1.24–1.36), while education showed no protective association. At the neighborhood level, urban residence (OR = 1.09, 95% CI: 1.01–1.17) and greater community socioeconomic disadvantage were independently associated with higher T2DM risk. Neighborhood factors accounted for 32.2% of the total variance, and moving to a higher‐risk neighborhood was associated with a 3.29‐fold increase in median odds. Socioeconomic contexts at both individual and contextual levels independently shape T2DM risk in LMICs, underscoring the importance of place‐based public health strategies alongside individual‐level interventions.

## Graphical abstract

Across 14 low‐ and middle‐income countries (1,090,328 individuals; 38,174 neighborhoods), higher individual wealth increased type 2 diabetes mellitus odds (OR = 1.30). Urban residence and community socioeconomic disadvantage independently elevated risk after controlling for individual factors. Neighborhood‐level determinants explained 32.2% of total variance in diabetes risk, among the largest contextual effects reported in this setting. Place‐based public health interventions, alongside individual‐level strategies, are essential to address the growing diabetes epidemic in LMICs.

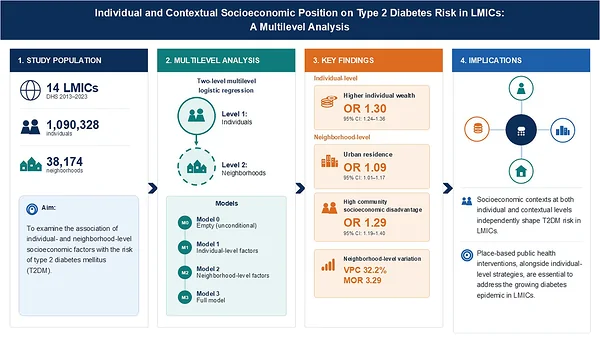

## 1 Introduction

According to the World Health Organization (WHO), type 2 diabetes mellitus (T2DM) is one of the fastest‐growing public health challenges in the world, particularly in low‐ and middle‐income countries (LMICs) [1]. As populations in such areas continue to move to rapidly expanding cities, change their diets, and undergo economic transitions, the burden of T2DM has increased across many settings. T2DM is characterized by insulin resistance and persistent hyperglycemia [2, 3]. Up to 60% of individuals living with T2DM die from one or more associated complications, primarily heart attack or stroke, but also chronic kidney failure, neuropathy (simple ideational degeneration that produces pain or numbness in an extremity), and necrobiosis lipoidica (a degenerative skin disease with blood vessel changes) [1, 3]. The WHO projects that overall worldwide numbers of people with T2DM will continue to rise substantially in the coming decades and virtually all new cases will be found in LMICs [2].

The rise of T2DM in LMICs cannot be attributed solely to genetic predispositions or personal behavior. It is closely linked with socioeconomic conditions at all levels, from individual characteristics like income and education [4] to neighborhood conditions like concentrated poverty, access to health services and design of the urban environment [5], and broader national‐level factors such as economic strength and health system infrastructure [6]. These factors mold health outcomes by affecting access to healthcare, diet, physical activity, and exposure to health hazards.

Urbanization has become a major driving force behind the diabetes epidemic in LMICs. While urban areas provide better access to health services and economic opportunities for households, people often end up living a sedentary lifestyle and turn to consuming more energy‐dense processed foods [7]. This *urban diabetes syndrome* is an example of how urbanization, as a process of development and also a noncommunicable disease risk factor, can lead to dual outcomes [8, 9].

Socioeconomic position (SEP) influences diabetes risk through a variety of routes. At the individual level, higher levels of education and income may improve health literacy and access to preventive healthcare. However, in many LMICs, these advantages are also associated with service‐sector and other white‐collar occupations that involve lower levels of physical activity, more sedentary lifestyles, and increased exposure to energy‐dense diets, which may increase the risk of T2DM [10]. Conversely, in many LMIC settings, individuals with lower socioeconomic status may have reduced access to energy‐dense processed foods and are more likely to engage in physically demanding daily activities, which may contribute to a lower risk of T2DM. However, they may still face important barriers in accessing healthcare and preventive services [7]. Neighborhood‐level factors such as community poverty rates and access to public services are another aspect where the influence of broader societal and physical environments compound these risks [11, 12].

Despite the growing body of research on socioeconomic determinants of T2DM, existing evidence from LMICs remains limited and heterogeneous, particularly with respect to multilevel analyses that simultaneously examine individual and neighborhood‐level factors. While some studies have examined the association between area‐level socioeconomic deprivation and diabetes in specific settings, including neighborhood‐level and regional analyses, comprehensive cross‐national studies that simultaneously integrate contextual and compositional factors remain scarce [5, 10, 13, 14]. This neglect is significant, as neighborhoods shape the socioeconomic environments in which individuals live, influencing their access to resources, opportunities, and health‐related behaviors over the life course.

Without a comprehensive multilevel understanding of the socioeconomic determinants of T2DM, it will be difficult to design and implement effective prevention and control strategies that address both individual and contextual risk factors. Much of the existing research has focused predominantly on individual compositional factors associated with T2DM in LMICs, with established evidence that individual‐level characteristics such as age, sex, and body mass index (BMI) are associated with diabetes risk [1, 2]. However, far less attention has been paid to how neighborhood‐level socioeconomic characteristics might independently influence T2DM risk or modify the effects of individual‐level factors.

The present study endeavors to address this gap by developing and testing a comprehensive multilevel model of socioeconomic factors associated with T2DM risk in LMICs. By simultaneously examining individual‐ and neighborhood‐level socioeconomic characteristics, we aim to answer the following research questions. (1) How do individual‐level socioeconomic factors (wealth, education, occupation) influence T2DM risk in LMICs? (2) To what extent do neighborhood‐level socioeconomic factors (urban residence, community disadvantage) independently affect T2DM risk after controlling for individual characteristics? (3) How much of the variation in T2DM risk among individuals is conditioned by differences between neighborhoods? (4) Do the relationships between SEP and T2DM risk vary across different countries and regions? This multilevel approach is essential for informing targeted and effective public health interventions that address the growing burden of diabetes in LMICs, considering both individual and contextual determinants of health.

## 2 Methods

### 2.1 Study Design and Data Sources

The Demographic and Health Surveys (DHSs), which are nationally representative household surveys carried out in LMIC nations, provided the data for this cross‐sectional analysis [15]. Data from 14 recent DHSs conducted between 2013 and 2023 were analyzed, including surveys from Benin, Gambia, Kenya, Lesotho, Namibia, South Africa, Zambia, Bangladesh, India, Philippines, Timor‐Leste, Albania, Tajikistan, and Haiti. The study's generalizability is improved by this regional diversity, which captures a variety of environmental, cultural, and economic factors. Households are the sampling unit in the DHS's multistage, stratified sampling design. Within each sample household, all women and men meeting the eligibility criteria are interviewed. Because the surveys are not self‐weighting, weights are calculated to account for unequal selection probabilities as well as for nonresponse. With weights applied, survey findings represent the full target populations. Household, women's, and, in the majority of nations, men's questionnaires are all included in the DHSs. Every country uses the same interviewer training, oversight, and implementation procedures for all three DHS questions. The study employed structured questionnaires and household interviews to gather information on socioeconomic factors, biomarker measurements, and health habits.

### 2.2 Sampling Technique

DHS uses a stratified, multistage sampling design to guarantee national representativeness. Using a probabilistic approach that considers the distribution of urban and rural areas, families serve as the primary sampling units. All participants were eligible individuals who resided in each of the selected households and were at least 18 years old. The results were adjusted for nonresponses and differential selection probabilities using sampling weights to ensure that they accurately represented the target population. The pooled dataset comprised 1,090,328 respondents from 38,174 neighborhoods across 14 countries.

The sample size was not predetermined, as this study was based on secondary analysis of existing DHS data. All eligible participants from the selected surveys were included in the analysis. Of the 1,090,328 observations, 123,088 had missing T2DM outcome data, 17,121 had missing age, education, wealth, and neighborhood identifier data; 283,658 had missing BMI data; and 10,336 had missing smoking data. Some observations had missing data for more than one variable. No missing values were observed for sex, professional occupation, urban residence, or community socioeconomic disadvantage. Complete‐case analysis resulted in 766,272 observations in the fully adjusted model.

### 2.3 Outcome Variable

The main outcome variable of this study was T2DM, defined using a combination of self‐reported diagnosis and fasting blood glucose (FBG) levels. Blood glucose was measured using the HemoCue 201+ analyzer after at least 8 h of fasting. T2DM was defined as FBG ≥126 mg/dL (7.0 mmol/L) or a self‐reported previous diagnosis by a health professional, in accordance with established diagnostic criteria [1]. Individuals with FBG levels between 110 and 125 mg/dL (6.1–6.9 mmol/L), classified as prediabetes, were excluded from the analysis. Nonfasting glucose measurements were not used, as DHS biomarker data are based on standardized fasting protocols.

### 2.4 Independent Variables

#### 2.4.1 Individual‐Level Factors

The multilevel models included the following individual‐level factors: respondents’ age (categorical, grouped into three categories: <25 years [young adults, reference], 25–34 years [adults], and ≥35 years [middle‐aged/older adults]); educational attainment (binary: secondary education or higher vs. less than secondary); gender (binary: female vs. male); professional employment status (binary: professional vs. nonprofessional); and behavioral and health‐related variables, such as BMI (categorical: underweight <18.5 kg/m^2^, normal weight 18.5–24.9 kg/m^2^ [reference], overweight 25.0–29.9 kg/m^2^, and obese ≥30.0 kg/m^2^, based on WHO standard cut‐offs [16]), and smoking status (binary: current smoker vs. nonsmoker).

A binary wealth indicator (richer), which divided people into higher and lower wealth groups, was used to measure socioeconomic status. Since DHS does not collect direct data on household income or expenditure, this was derived from the DHS wealth index, a validated representative for household economic status. Principal component analysis of household assets and housing characteristics, such as the number of rooms and possession of heating devices, are used to create the index. Additional methodological information can be found elsewhere [17, 18].

The involvement in professionally skilled (white‐collar) occupations was specifically examined in relation to employment status. Unlike blue‐collar jobs, which are manual labor‐intensive, white‐collar jobs are usually classified as professional, managerial, or administrative roles. Social standing and human capital are both reflected in employment status and educational attainment.

Survey year (represented as categorical dummy variables) and geographic region (categorical) were also included to account for temporal and geographic variability.

#### 2.4.2 Neighborhood‐Level Factors

The following community‐level covariates were adjusted in the models: area of residence (binary: urban vs. rural) and community‐level socioeconomic disadvantage. We referred to clusters in the same primary sampling unit (PSU), as defined in the DHS, as the neighborhood. The community disadvantage score was developed by summing the individual‐level variables within each cluster, specifically the proportion of individuals with no education, unemployment, and poverty (measured as the lowest two wealth quintiles in the DHS). These components were then aggregated into an overall socioeconomic score for each area. Equal weighting was applied to each component, consistent with previous DHS‐based multilevel studies, due to the absence of a priori evidence supporting differential weighting.

Community socioeconomic disadvantage was treated as a categorical variable with three levels: least disadvantaged (reference), moderately disadvantaged, and highly disadvantaged. These categories were based on tertiles of the composite community disadvantage score, providing a clear distinction across levels of socioeconomic disadvantage. This approach is consistent with previous DHS‐based multilevel analyses of contextual socioeconomic factors [17].

### 2.5 Statistical Analyses

#### 2.5.1 Descriptive Analyses

Descriptive statistics summarize key variables by providing counts, means, standard deviations, and percentages. Multilevel logistic regression models were used to analyze the association between socioeconomic factors and the risk of T2DM, considering the hierarchical structure of the data.

#### 2.5.2 Modeling Approaches

**FIGURE 1:**
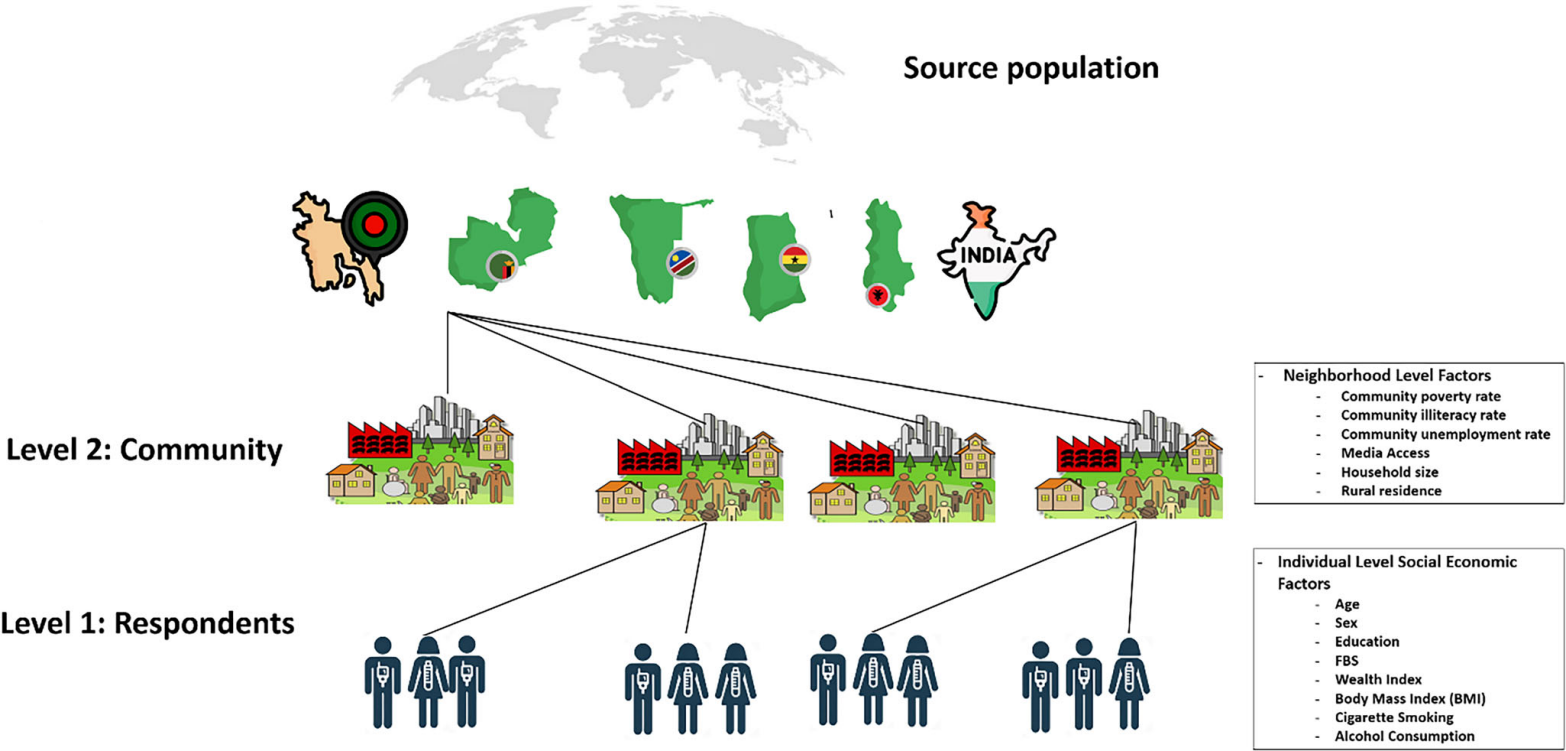
Source population modeling, individuals were at level 1, and neighborhoods were at level 2.

We used multilevel logistic regression models to evaluate associations of individual, compositional, and contextual factors related to diabetes [18]. We proposed a two‐level model for the binary outcome of diabetes status (presence or absence of diabetes). In this model, individuals were at level 1, and neighborhoods were at level 2 [19, 20] (see Figure 1).

We created a total of four models. Model 0 was an empty (unconditional) model, which did not include any explanatory variables. Model 1 consisted of only individual‐level factors, and model 2 contained only neighborhood‐level factors. Model 3, the full model, used simultaneous controls for both individual‐ and neighborhood‐level factors.

#### 2.5.3 Fixed Effects (Measures of Association)

Results of fixed effects models were presented as odds ratios (ORs) with 95% confidence intervals (CIs).

#### 2.5.4 Random Effects (Measures of Variation)

Potential contextual effects were evaluated using intraclass correlation (ICC) and median odds ratio (MOR). Within‐country ICC was used to assess the degree of similarity between respondents living in the same neighborhood. The ICC shows the proportion of variance in the total reported probability of diabetes that is explained by the neighborhood level, which is equivalent to the degree of clustering of the odds of reporting diabetes within the neighborhood level. A linear threshold approach over latent variables was used to estimate it.

Utilizing the Larsen et al. [21] framework, we described the random makes‐in odds at the local effector center. The MOR provides an odds ratio and the share of the likelihood of having diabetes attributed to a neighbor context by quantifying variance at the second or third level. When neighborhood‐level effects are added, the model is not improved, with an MOR of 1. On the other hand, an increased MOR signifies that the determinants at the individual level are less important than the factors at an aggregate level in explaining the likelihood of having diabetes.

#### 2.5.5 Model Fit and Specifications

Analyses were performed using the MLwiN software, v. 2.31. Markov Chain Monte Carlo process was used to estimate parameters [22, 23].

#### 2.5.6 Meta‐Analyses

To complement the multilevel modeling approach and further contextualize findings across countries, we conducted a series of meta‐analyses using random‐effects models. These analyses aimed to: (1) estimate the pooled prevalence of T2DM across the 14 countries studied; (2) quantify the consistency of associations between key socioeconomic factors and T2DM risk; and (3) examine potential heterogeneity in these relationships across different regions.

For the prevalence meta‐analysis, we calculated the prevalence of T2DM for each country and pooled these estimates using a random‐effects restricted maximum likelihood model to account for between‐country heterogeneity. For the association meta‐analyses, we examined relationships between T2DM and four key factors: (1) occupational status (white‐collar vs. non‐white‐collar); (2) educational attainment (highly educated vs. not highly educated); (3) urban versus rural residence; and (4) community socioeconomic disadvantage (least vs. most disadvantaged).

Each country provided a study‐level estimate weighted by the inverse of its variance, and heterogeneity was assessed using *I* ^2^ statistics, with values of 25%, 50%, and 75% considered as low, moderate, and high heterogeneity, respectively. We also calculated τ^2^ to quantify between‐study variance and conducted tests for group differences (Qb statistics) to determine whether the associations varied significantly by geographic region. Forest plots were generated to visually represent the country‐specific and pooled effect sizes.

These meta‐analyses complemented our multilevel models by providing additional insights into the consistency and variability of socioeconomic determinants of T2DM across different countries and regions, thereby strengthening the robustness and generalizability of our findings.

## 3 Results

### 3.1 Sample Characteristics

The pooled dataset included data from 1,090,328 individuals across 38,174 neighborhoods in 14 LMICs. Table 1 presents the sociodemographic characteristics of the study population. The sample comprised predominantly female participants (85.5%, *n* = 827,113) compared to males (14.5%, *n* = 140,127). Age distribution showed 32.6% (*n* = 315,064) young adults, 42.7% (*n* = 412,595) adults, and 24.6% (*n* = 237,855) middle‐aged/older adults. Approximately two‐thirds of participants (63.5%, *n* = 692,563) had attained secondary or higher education, while 36.5% (*n* = 397,765) reported no secondary education. Regarding socioeconomic indicators, 38.1% (*n* = 415,154) of participants were classified in the higher wealth quintiles, while 61.9% (*n* = 675,174) belonged to lower wealth categories. The vast majority of participants (97.5%, *n* = 1,063,020) were not employed in white‐collar occupations, with only 2.5% (*n* = 27,308) reporting professional employment. Analysis of health‐related characteristics revealed that 59.3% (*n* = 478,122) of participants had normal BMI, while 17.1% (*n* = 137,814) were underweight, 17.4% (*n* = 139,998) were overweight, and 6.3% (*n* = 50,736) were classified as obese. Smoking prevalence was notably low, with only 2.2% (*n* = 23,718) of participants reporting current smoking compared to 97.8% (*n* = 1,056,274) nonsmokers. The distribution of participants across residential settings showed that 27.4% (*n* = 264,687) resided in urban areas, while 72.6% (*n* = 702,553) lived in rural areas. When examining neighborhood socioeconomic characteristics, participants were almost equally distributed across least disadvantaged (33.8%, *n* = 368,378), moderately disadvantaged (32.9%, *n* = 358,770), and highly disadvantaged (33.3%, *n* = 363,180) neighborhoods.

**TABLE 1:** Summary of pooled sample characteristics of the Demographic and Health Surveys data in Africa, the Americas, South‐East Asia, Europe, and the Western Pacific regions, 2013–2023.

Individual‐level factors		Number (%)
Sex	Male	140,127 (14.5)
	Female	827,113 (85.5)
Age	Young adult	315,064 (32.6)
	Adult	412,595 (42.7)
	Middle‐age/older adult	237,855 (24.6)
Secondary or higher education	No	397,765 (36.5)
	Yes	692,563 (63.5)
Richer versus others	No	675,174 (61.9)
	Yes	415,154 (38.1)
White collar versus others	No	1,063,020 (97.5)
	Yes	27,308 (2.5)
BMI	Underweight	137,814 (17.1)
	Normal	478,122 (59.3)
	Overweight	139,998 (17.4)
	Obese	50,736 (6.3)
Smoking	Not smoking	1,056,274 (97.8)
	Smoking	23,718 (2.2)

**Neighborhood‐level factors**		
Place of residence	Urban	264,687 (27.4)
	Rural	702,553 (72.6)
Community socioeconomic disadvantage	Least	368,378 (33.8)
	Moderate	358,770 (32.9)
	High	363,180 (33.3)

### 3.2 Cross‐National Variations in T2DM Prevalence

**FIGURE 2:**
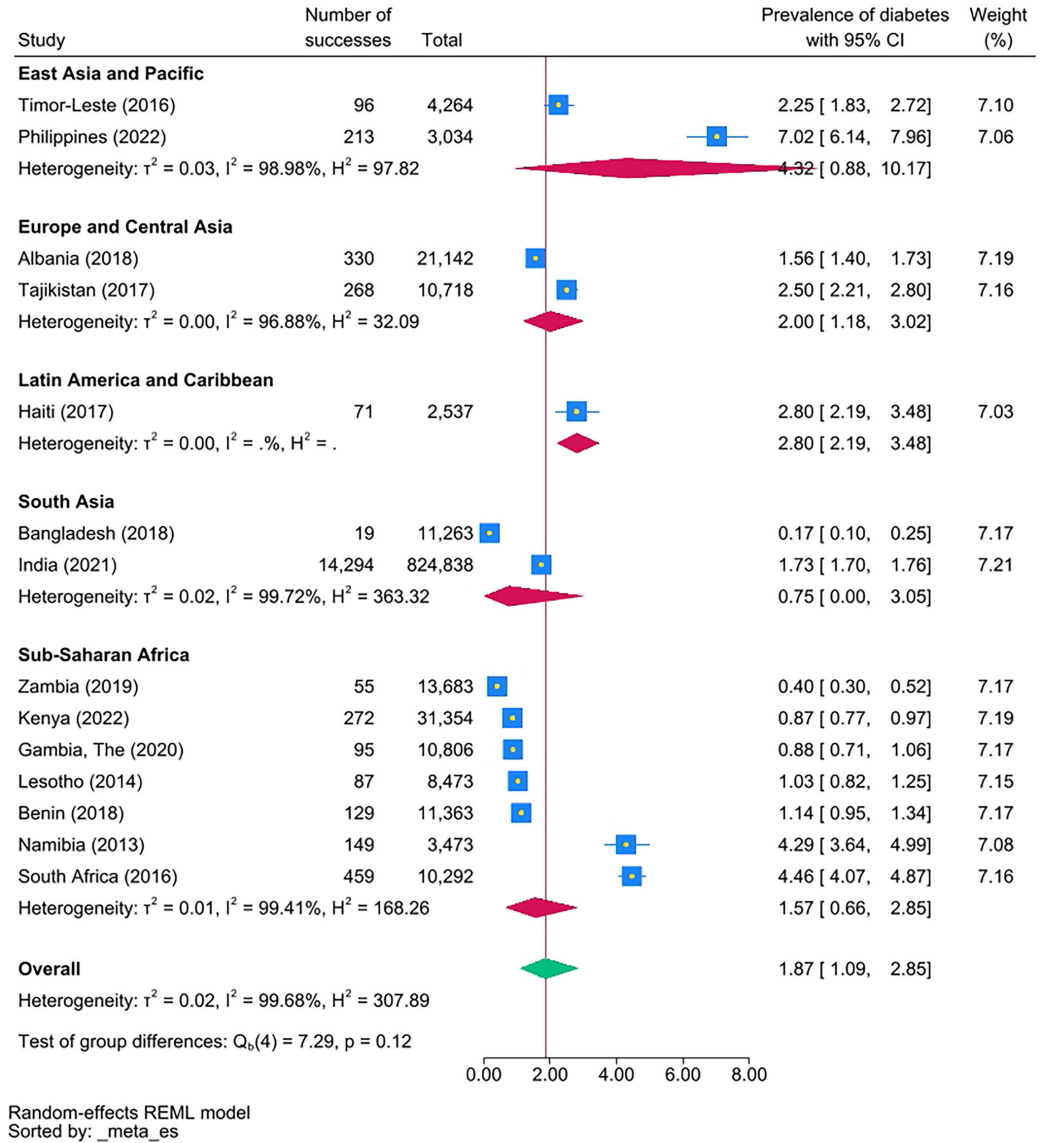
Forest plot showing pooled prevalence of type 2 diabetes mellitus (T2DM) across 14 LMICs with 95% CI.

The meta‐analysis of T2DM prevalence across 14 LMICs revealed substantial heterogeneity in disease burden (Figure 2). The overall pooled prevalence was estimated at 1.87% (95% CI: 1.09−2.85), though this aggregate estimate masks considerable variation across regions and individual countries. Distinct regional patterns emerged in our analysis, highlighting the geographically varied nature of the diabetes epidemic:

East Asia and Pacific: Countries in this region showed divergent prevalence rates, with the Philippines demonstrating one of the highest T2DM prevalence rates in our sample at 7.02% (95% CI: 6.14−7.96), while Timor‐Leste reported a significantly lower rate of 2.25% (95% CI: 1.83−2.72). The pooled regional estimate was 4.32% (95% CI: 0.88−10.17), with substantial heterogeneity observed (*I* ^2^ = 98.98%).

Europe and Central Asia: Moderate prevalence rates were found in Europe and Central Asia, with Tajikistan showing 2.50% (95% CI: 2.21−2.80) and Albania reporting 1.56% (95% CI: 1.40−1.73). The regional estimate of 2.00% (95% CI: 1.18−3.02) reflected less heterogeneity than other regions, though differences remained notable (*I* ^2^ = 96.88%).

Latin America and Caribbean: Haiti, the sole representative from this region, demonstrated an intermediate prevalence rate of 2.80% (95% CI: 2.19−3.48), slightly above the global average.

South Asia: South Asia displayed the widest intraregional disparity, with Bangladesh reporting the lowest prevalence in our entire sample at merely 0.17% (95% CI: 0.10−0.25), while India showed a moderate rate of 1.73% (95% CI: 1.70−1.76). The regional pooled estimate was 0.75% (95% CI: 0.00−3.05), with extremely high heterogeneity (*I* ^2^ = 99.72%).

Sub‐Saharan Africa: Sub‐Saharan Africa exhibited the most varied diabetes landscape, with prevalence ranging from as low as 0.40% in Zambia (95% CI: 0.30−0.52) to markedly higher rates in South Africa at 4.46% (95% CI: 4.07−4.87) and Namibia at 4.29% (95% CI: 3.64−4.99). Other countries in the region, including Kenya, Gambia, Lesotho, and Benin, showed relatively low prevalence ranging from 0.87% to 1.14%. The regional estimate was 1.57% (95% CI: 0.66−2.85), with considerable heterogeneity (*I* ^2^ = 99.41%).

### 3.3 Association Between Occupational Status and T2DM

**FIGURE 3:**
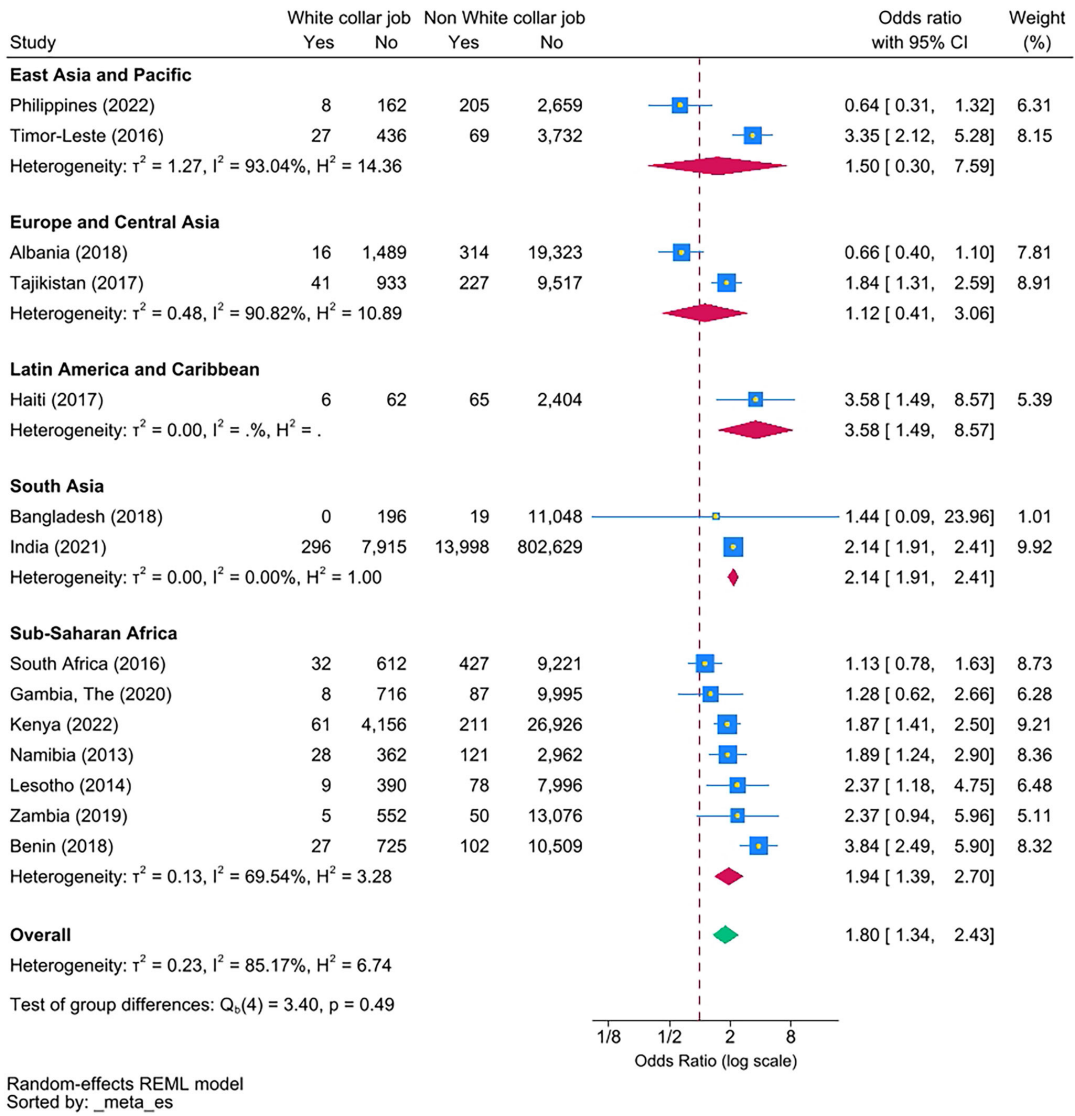
Association between white‐collar employment and T2DM prevalence by country (odds ratios with 95% CI).

Our meta‐analysis examining the relationship between occupational status and T2DM risk revealed a significant positive association between white‐collar employment and diabetes (Figure 3). The overall pooled odds ratio was 1.80 (95% CI: 1.34−2.43), indicating that individuals in white‐collar occupations had approximately 80% higher odds of T2DM compared to those in non‐white‐collar jobs.

The strength and direction of this association varied considerably across regions: Sub‐Saharan Africa showed a consistent positive association (pooled OR = 1.94, 95% CI: 1.39−2.70), with particularly strong effects in Benin (OR = 3.84, 95% CI: 2.49−5.90) and moderate effects in other countries. South Asia demonstrated a positive association, primarily driven by India (OR = 2.14, 95% CI: 1.91−2.41), though Bangladesh had insufficient white‐collar cases for reliable estimation. Latin America and the Caribbean, represented by Haiti, showed the strongest regional association (OR = 3.58, 95% CI: 1.49−8.57). Europe and Central Asia displayed mixed results, with Tajikistan showing a positive association (OR = 1.84, 95% CI: 1.31−2.59), while Albania showed a nonsignificant protective effect (OR = 0.66, 95% CI: 0.40−1.10). East Asia and Pacific exhibited the most heterogeneity (*I* ^2^ = 93.04%), with Timor‐Leste showing a strong positive association (OR = 3.35, 95% CI: 2.12−5.28), while the Philippines showed a nonsignificant inverse relationship (OR = 0.64, 95% CI: 0.31−1.32).

### 3.4 Association Between Educational Attainment and T2DM

**FIGURE 4:**
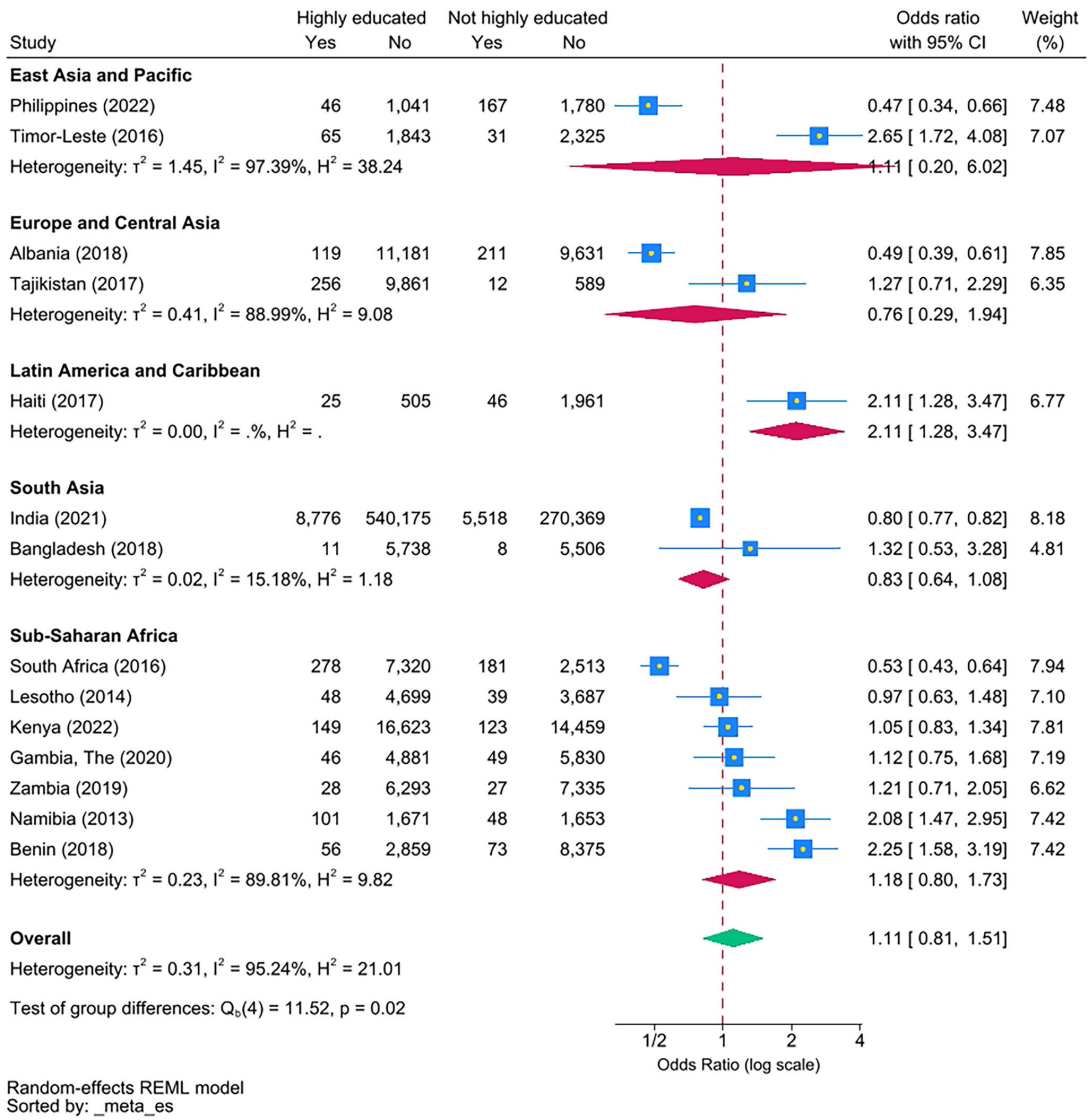
Odds of T2DM comparing higher versus lower education across countries in LMICs.

Our meta‐analysis examining the relationship between higher educational attainment and T2DM risk revealed considerable heterogeneity across regions (Figure 4). Overall, the pooled odds ratio was 1.11 (95% CI: 0.81−1.51), suggesting no statistically significant association between higher education and diabetes risk across all LMICs studied.

The relationship between education and T2DM varied significantly by region (test for group differences: Qb(4) = 11.52, *p* = 0.02): Sub‐Saharan Africa demonstrated marked intraregional variability, with South Africa showing a protective effect (OR = 0.53, 95% CI: 0.43−0.64), while Namibia (OR = 2.08, 95% CI: 1.47−2.95) and Benin (OR = 2.25, 95% CI: 1.58−3.19) showed significantly higher T2DM risk among the highly educated. East Asia and Pacific exhibited extreme heterogeneity (*I* ^2^ = 97.39%), with opposite effects in the Philippines (protective: OR = 0.47, 95% CI: 0.34−0.66) versus Timor‐Leste (risk‐enhancing: OR = 2.65, 95% CI: 1.72−4.08). Europe and Central Asia showed similar divergence, with Albania demonstrating a protective association (OR = 0.49, 95% CI: 0.39−0.61), while Tajikistan showed a nonsignificant positive association (OR = 1.27, 95% CI: 0.71−2.29). Latin America and the Caribbean, represented by Haiti, showed a significant positive association (OR = 2.11, 95% CI: 1.28−3.47). South Asia exhibited a nonsignificant pooled effect (OR = 0.83, 95% CI: 0.64−1.08), with India showing a slight protective association (OR = 0.80, 95% CI: 0.77−0.82).

### 3.5 Urban Versus Rural Residence and Diabetes Risk

**FIGURE 5:**
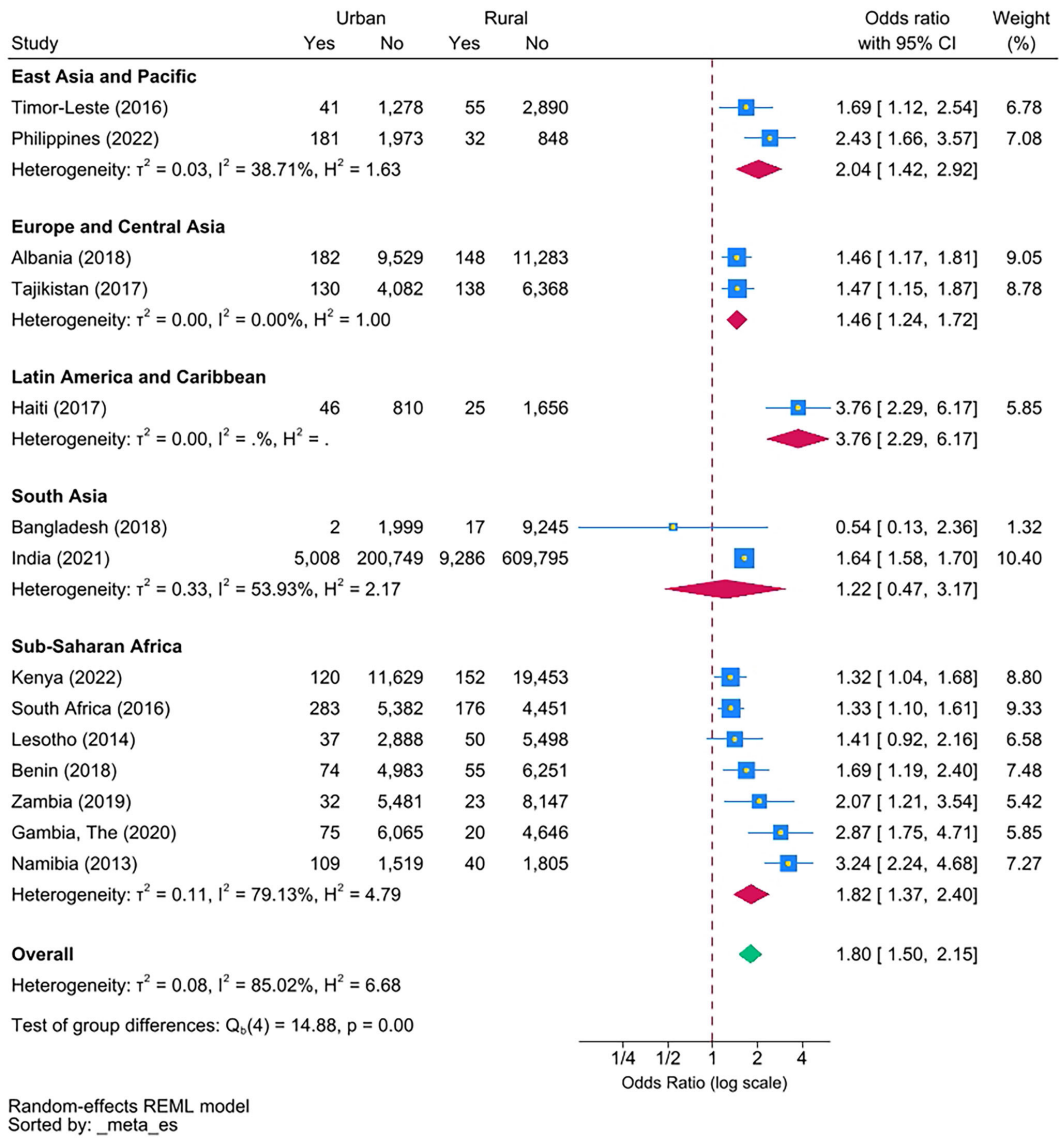
Risk of T2DM associated with urban residence versus rural across 14 LMICs.

Our meta‐analysis examining the association between urban residence and T2DM risk revealed a strong, consistent relationship across most countries studied (Figure 5). The overall pooled odds ratio was 1.80 (95% CI: 1.50−2.15), indicating that urban residents had 80% higher odds of T2DM compared to their rural counterparts.

The association between urban residence and diabetes risk varied significantly by region (test for group differences: Qb(4) = 14.88, *p* < 0.001): Latin America and the Caribbean, represented by Haiti, showed the strongest urban−rural disparity (OR = 3.76, 95% CI: 2.29−6.17). East Asia and Pacific demonstrated a clear urban disadvantage (pooled OR = 2.04, 95% CI: 1.42−2.92), with both the Philippines (OR = 2.43, 95% CI: 1.66−3.57) and Timor‐Leste (OR = 1.69, 95% CI: 1.12−2.54) showing significant effects. Sub‐Saharan Africa exhibited consistent positive associations (pooled OR = 1.82, 95% CI: 1.37−2.40), with particularly pronounced effects in Namibia (OR = 3.24, 95% CI: 2.24−4.68) and Gambia (OR = 2.87, 95% CI: 1.75−4.71). Europe and Central Asia showed remarkably consistent results with minimal heterogeneity (*I* ^2^ = 0.00%), with both Albania (OR = 1.46, 95% CI: 1.17−1.81) and Tajikistan (OR = 1.47, 95% CI: 1.15−1.87) demonstrating similar urban disadvantages. South Asia showed mixed results (pooled OR = 1.22, 95% CI: 0.47−3.17), with India showing a significant urban disadvantage (OR = 1.64, 95% CI: 1.58−1.70), while Bangladesh showed a nonsignificant inverse relationship (OR = 0.54, 95% CI: 0.13−2.36).

### 3.6 Association Between Community Socioeconomic Disadvantage and T2DM

**FIGURE 6:**
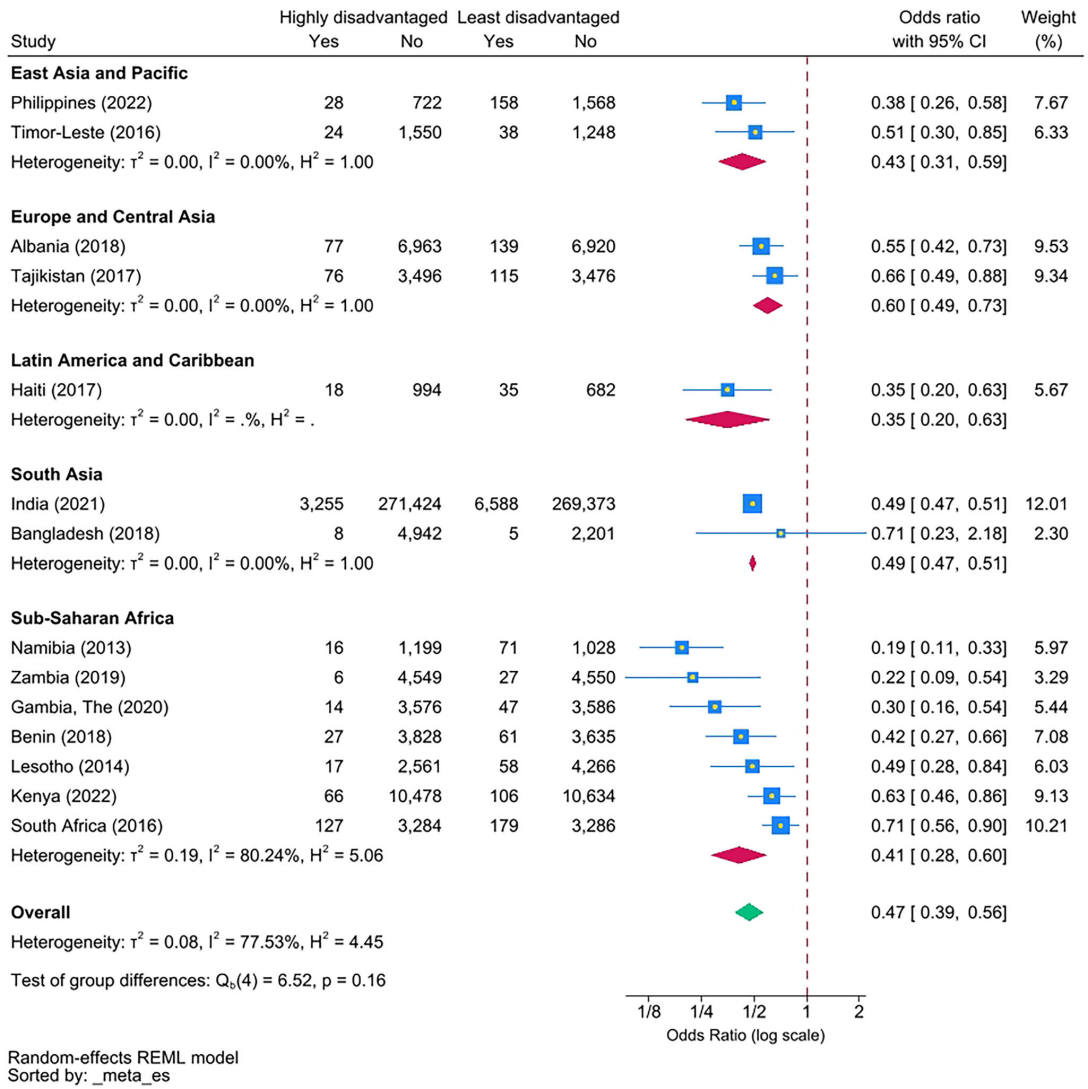
Association between high versus low community disadvantage and odds of T2DM across LMICs.

Our meta‐analysis examining the association between neighborhood socioeconomic advantage and T2DM risk revealed a consistent pattern across countries (Figure 6). The overall pooled odds ratio was 2.14 (95% CI: 1.77−2.58), indicating that individuals living in less disadvantaged (more affluent) neighborhoods had more than twice the odds of developing T2DM compared to those in more disadvantaged areas. Note that for the meta‐analysis, the reference category is the most disadvantaged group, so the OR of 2.14 reflects higher odds among the least disadvantaged (most affluent) neighborhoods, consistent with the nutrition‐transition pattern whereby greater community affluence is initially associated with increased T2DM risk. In the multilevel fixed‐effects models (Table 2), the reference is the least disadvantaged group, yielding an OR of 1.29 for highly disadvantaged neighborhoods. These estimates derive from different analyses and show associations in opposite directions; they should therefore be interpreted separately.

**TABLE 2:** Individual compositional and contextual factors associated with diabetes identified by multivariable multilevel logistic regression models, Demographic and Health Surveys data, 2013–2023.

	Model 0^a^ OR (95 % CI)	Model 1^b^ OR (95 % CI)	Model 2^c^ OR (95 % CI)	Model 3^d^ OR (95 % CI)
**Measures of associations (fixed effects model)**				
**Traditional risk factors**				
Age				
Young adult		1 (reference)		1 (reference)
Adult		2.31 (2.16−2.47)		2.28 (2.14−2.44)
Middle‐age/older adult		6.92 (6.46−7.40)		6.78 (6.34−7.27)
BMI				
Underweight		0.72 (0.70−0.77)		0.73 (0.68−0.78)
Normal		1 (reference)		1 (reference)
Overweight		1.55 (1.49−1.62)		1.53 (1.47−1.60)
Obese		2.52 (2.39−2.66)		2.46 (2.33−2.60)
Smoking versus nonsmoker		1.60 (1.25−2.05)		1.57 (1.23−2.01)
**Individual‐level of socioeconomic factors**				
Education: secondary+ versus others		1.03 (0.99−1.08)		0.99 (0.95−1.04)
Richer household's versus others		1.42 (1.36–1.48)		1.30 (1.24−1.36)
White‐collar jobs versus others		1.17 (1.00−1.37)		1.16 (0.99−1.35)
**Neighborhood‐level socioeconomic factors**				
Urban versus rural			1.22 (1.15−1.29)	1.09 (1.01−1.17)
Community socioeconomic disadvantage				
Highly disadvantaged			1.74 (1.63−1.86)	1.29 (1.19−1.40)
Moderately disadvantaged			1.38 (1.31−1.45)	1.15 (1.08−1.23)
Least disadvantaged			1 (reference)	1 (reference)
**Measures of variations (random effects)**				
Variance (95% CI)	1.32 (1.27−1.37)	1.54 (1.47−1.60)	1.28 (1.23−1.33)	1.56 (1.50−1.62)
VPC (%)	28.6	31.8	28.0	32.2
MOR (95% CI)	2.99	3.26	2.94	3.29

*Note*: To investigate the factors influencing the outcome at the individual and community levels, multilevel logistic regression models were fitted. Wealth status (richer vs. poorest), educational attainment (secondary or higher vs. none), career type (professional vs. nonprofessional), sex (female vs. male), age groups (25–34 years, 35+ years; reference category <25 years), body mass index (underweight, overweight, obese; reference category normal weight), smoking status (smoker vs. nonsmoker), survey year, and place of residence were among the predictors at the individual level. The models did not include employment status (working vs. not working) as a predictor. The least disadvantaged communities served as the reference, and predictors at the community level included socioeconomic disadvantage, which was divided into moderate and high levels, and place of residence (rural vs. urban).

Abbreviations: BMI, body mass index; CI, confidence interval; MOR, median odds ratio; OR, odds ratio; VPC, variance partition coefficient.

^a^ Model 0—empty null model, baseline model without any explanatory variables (unconditional model).

^b^ Model 1—adjusted for only individual‐level factors.

^c^ Model 2—adjusted for only neighborhood‐level factors.

^d^ Model 3—adjusted for individual‐ and neighborhood‐level factors (full model).

This association was remarkably consistent across regions (test for group differences: Qb(4) = 6.52, *p* = 0.16): Sub‐Saharan Africa showed the most pronounced intraregional variation (*I* ^2^ = 80.24%), with particularly strong associations in Namibia (OR = 5.18, 95% CI: 2.99−8.96) and Zambia (OR = 4.50, 95% CI: 1.86−10.91), while South Africa showed a more modest effect (OR = 1.41, 95% CI: 1.12−1.78). East Asia and Pacific demonstrated consistent positive associations with minimal heterogeneity (*I* ^2^ = 0.00%), with both the Philippines (OR = 2.60, 95% CI: 1.72−3.92) and Timor‐Leste (OR = 1.97, 95% CI: 1.17−3.30) showing significant effects. Latin America and the Caribbean, represented by Haiti, showed a strong positive association (OR = 2.83, 95% CI: 1.59−5.05). Europe and Central Asia exhibited consistent results with no heterogeneity (*I* ^2^ = 0.00%), with both Albania (OR = 1.82, 95% CI: 1.37−2.40) and Tajikistan (OR = 1.52, 95% CI: 1.13−2.04) showing significant associations. South Asia results were primarily driven by India (OR = 2.04, 95% CI: 1.95−2.13), with Bangladesh showing a nonsignificant positive association (OR = 1.40, 95% CI: 0.46−4.29).

### 3.7 Measures of Associations (Fixed Effects Model)

The results of the multilevel logistic regression models, which evaluated the associations between socioeconomic factors and the risk of T2DM at individual and neighborhood levels, are presented in Table 2. In the fully adjusted model (model 3), individual‐level factors emerged as strong predictors of T2DM risk. Age demonstrated a pronounced gradient effect, with adults aged 25–34 years having more than twice the odds of T2DM compared to young adults (OR = 2.28, 95% CI: 2.14−2.44), while middle‐aged/older adults exhibited nearly seven times higher odds (OR = 6.78, 95% CI: 6.34−7.27).

BMI showed a clear dose−response relationship with T2DM risk. Compared to normal‐weight individuals, those who were underweight had lower odds (OR = 0.73, 95% CI: 0.68−0.78), while overweight (OR = 1.53, 95% CI: 1.47−1.60) and obese individuals (OR = 2.46, 95% CI: 2.33−2.60) demonstrated progressively higher odds of T2DM. Smoking was also significantly associated with increased diabetes risk (OR = 1.57, 95% CI: 1.23−2.01).

Among socioeconomic indicators at the individual level, higher household wealth was associated with increased T2DM odds (OR = 1.30, 95% CI: 1.24−1.36), while the association with white‐collar occupations was positive but marginally nonsignificant (OR = 1.16, 95% CI: 0.99−1.35). Higher educational attainment showed no protective effect against T2DM (OR = 0.99, 95% CI: 0.95−1.04).

At the neighborhood level, urban residence maintained a significant positive association with T2DM risk in the fully adjusted model, though the effect size diminished after controlling for individual factors (OR = 1.09, 95% CI: 1.01−1.17). Community socioeconomic disadvantage demonstrated a gradient relationship with T2DM risk, with highly disadvantaged (OR = 1.29, 95% CI: 1.19−1.40) and moderately disadvantaged neighborhoods (OR = 1.15, 95% CI: 1.08−1.23) showing higher odds of T2DM compared to least disadvantaged areas, even after adjusting for individual‐level factors.

### 3.8 Measures of Variations (Random Effects)

As shown in Table 2, in the fully adjusted model (model 3), there was substantial variation in the odds of having T2DM across neighborhoods (σ^2^ = 1.56, 95% CI: 1.50−1.62). According to the variance partition coefficients (VPCs), 32.2% of the total variance in the odds of having T2DM could be attributed to neighborhood‐level factors, indicating that nearly one‐third of the variation in diabetes risk was explained by contextual determinants beyond individual characteristics.

Results from the MORs confirmed the presence of significant neighborhood contextual effects shaping individual T2DM risk. From the fully adjusted model (model 3), it was estimated that if an individual moved from a neighborhood with lower diabetes risk to one with higher risk (while maintaining the same individual characteristics), the median increase in their odds of having T2DM would be 3.29‐fold. This substantial MOR highlights the powerful influence of residential contexts on diabetes outcomes, even after accounting for individual sociodemographic and traditional risk factors.

Notably, the inclusion of individual‐level factors in model 1 increased both the between‐neighborhood variance (1.54 vs. 1.32 in the empty model) and the MOR (3.26 vs. 2.99), suggesting that individual characteristics alone did not account for neighborhood clustering of diabetes risk. The neighborhood‐level model (model 2) showed lower variance (1.28) and MOR (2.94) compared to model 1, but the combined model (model 3) demonstrated the highest variance and MOR values, underscoring the complex, multilevel nature of diabetes risk determinants in LMICs.

## 4 Discussion

### 4.1 Main Findings

This study provides a comprehensive multilevel analysis of the socioeconomic determinants of T2DM in LMICs. Our meta‐analyses revealed significant heterogeneity in diabetes prevalence across countries, ranging from 0.17% in Bangladesh to 7.02% in the Philippines, reflecting diverse stages of the epidemiological transition. The relationship between education and diabetes varied significantly by region (*p* = 0.02), while urban residence consistently increased diabetes risk across different settings. These findings highlight both commonalities and variations in how socioeconomic factors influence diabetes risk across diverse LMIC contexts. Importantly, the added value of this study lies in simultaneously quantifying both individual‐level and neighborhood‐level socioeconomic influences on T2DM risk across multiple LMICs. This multilevel perspective enables a clearer distinction between compositional and contextual effects, which remains limited in the LMIC literature at this scale.

Our findings reveal complex patterns of association between socioeconomic factors and diabetes risk across individual and neighborhood levels. At the individual level, wealth demonstrated a positive association with T2DM risk, with individuals in the highest wealth quintile having 30% higher odds of diabetes compared to those in the lowest quintile (OR = 1.30, 95% CI: 1.24–1.36). This suggests that in the context of LMICs, greater economic resources may initially increase rather than decrease diabetes risk. Educational attainment beyond secondary level showed no significant protective effect against diabetes (OR = 0.99, 95% CI: 0.95–1.04). Professional occupation showed a positive but marginally nonsignificant association with diabetes risk (OR = 1.16, 95% CI: 0.99–1.35), suggesting that white‐collar employment may be associated with lifestyle factors that increase metabolic risk [24, 25].

At the neighborhood level, we found that urban residence was associated with higher T2DM risk (OR = 1.09, 95% CI: 1.01−1.17), even after adjusting for individual socioeconomic characteristics. This observed urban–rural difference may reflect broader environmental and structural conditions associated with urbanization, including greater exposure to sedentary lifestyles and changing dietary patterns; however, urban residence should be interpreted as a proxy indicator rather than a direct causal determinant [26, 27]. Community socioeconomic disadvantage demonstrated a clear gradient relationship with T2DM risk, with residents of highly disadvantaged neighborhoods having 29% higher odds of diabetes (OR = 1.29, 95% CI: 1.19−1.40) compared to those in the least disadvantaged areas. This finding highlights how contextual poverty and limited resources can independently increase diabetes risk beyond individual SEP.

Perhaps most striking was the substantial contextual effect we observed, with 32.2% of the total variation in T2DM risk attributable to neighborhood‐level factors. It is important to note that this 32.2% figure represents a VPC, a measure of contextual heterogeneity quantifying the proportion of total T2DM risk variation attributable to between‐neighborhood differences, rather than the effect size of any single predictor variable. The MOR of 3.29 indicates that if an individual moved from a neighborhood with low diabetes risk to one with a high risk (while maintaining identical individual characteristics), they would experience a median increase of 3.29 in their odds of having diabetes. This powerful neighborhood effect underscores the critical importance of place in shaping health outcomes in LMICs.

### 4.2 Comparison With Previous Studies

This study offers a unique multilevel analysis of how individual‐ and neighborhood‐level socioeconomic factors influence the risk of T2DM in LMICs. The results demonstrate how intricately individual traits like wealth, education, and occupation interact with broader community elements like urbanization and neighborhood socioeconomic disadvantage to shape diabetes risk.

At the individual level, wealth was linked to a higher risk of T2DM rather than a protective effect, with those in the wealthiest quintile having 30% higher odds of diabetes compared to those in the poorest quintile. This finding contradicts patterns typically observed in high‐income countries, where socioeconomic advantage often confers protection against chronic diseases [4, 28]. However, it aligns with the nutrition transition hypothesis proposed by Popkin et al. [29], suggesting that in rapidly developing economies, increased wealth initially translates to greater consumption of energy‐dense foods and reduced physical activity before health awareness and preventive behaviors can develop. Dinsa et al. [30] similarly found in their systematic review that in LMICs, obesity, a significant diabetes risk factor, is initially concentrated among the affluent before shifting to disadvantaged populations as countries develop economically.

Contrary to our expectations, education beyond secondary level did not demonstrate a significant protective effect against T2DM. This contrasts with evidence from high‐income countries, where higher educational attainment typically correlates with reduced diabetes risk [31, 32]. The absence of this protective association in our LMIC sample may reflect what Stringhini et al. [33] describe as contextual dependencies of education's health benefits—in settings where health literacy is generally low or where environmental constraints override individual knowledge, education's protective effects may be diminished. Additionally, as noted by Baker et al. [34], educational systems in many LMICs may not emphasize health education to the extent needed to translate into protective health behaviors.

Our finding of a positive but marginally nonsignificant association between professional occupation and T2DM risk aligns with emerging evidence from LMICs. For instance, Ali et al. [24] found that white‐collar workers in India had higher diabetes prevalence compared to manual laborers, attributed to more sedentary work environments and work‐related stress. This occupational pattern exemplifies what Ezzati et al. [25] term the early phase of the epidemiologic transition in developing countries, where chronic diseases initially affect the more affluent before shifting to disadvantaged groups.

At the neighborhood level, our results highlight the significant contextual influence on diabetes risk. Urban residence maintained a significant positive association with T2DM in the fully adjusted model, though the effect size diminished after controlling for individual factors. This finding supports the concept of urban diabetes described by Gassasse et al. [26] in their systematic review, which found consistently higher diabetes prevalence in urban areas of LMICs. The urban disadvantage likely stems from what Allender et al. [27] describe as obesogenic environments that promote motorized transportation, sedentary lifestyles, and greater availability of processed foods.

Particularly noteworthy was our finding that community socioeconomic disadvantage was associated with higher T2DM risk, with residents of highly disadvantaged neighborhoods having 29% higher odds of diabetes even after controlling for individual SEP. This echoes findings from Dagenais et al. [35] in the Prospective Urban Rural Epidemiology (PURE) study, which demonstrated that neighborhood deprivation independently influenced cardiovascular risk factors, including diabetes, across multiple LMICs. The mechanisms likely involve what Christine et al. [36] describe as contextual exposures, limited access to healthy food options, fewer recreational facilities, higher stress levels, and poorer healthcare access in disadvantaged communities.

Perhaps most striking was our finding that 32.2% of the total variation in T2DM risk was attributable to neighborhood‐level factors, with an MOR of 3.29. This indicates that if a person moved from a neighborhood with low diabetes risk to one with high risk (while maintaining the same individual characteristics), they would experience, on average, a 3.29‐fold increase in their odds of developing T2DM. This substantial contextual effect exceeds what has been found in high‐income countries; for instance, Laraia et al. [37] reported neighborhood factors accounting for only 11% of diabetes variation in a US‐based study. The stronger neighborhood influence we observed supports Macintyre and Ellaway's [38] contention that in resource‐constrained settings, contextual factors may exert more powerful effects on health than in affluent societies where individual resources can buffer environmental disadvantages.

### 4.3 Implications for Policy and Future Research

Our findings have substantial implications for public health policy and future research directions, particularly in the context of addressing the growing burden of T2DM in LMICs. The significant multilevel determinants of diabetes risk we identified call for equally multilevel intervention approaches that address both individual and contextual factors. These findings indicate that T2DM prevention in LMICs cannot rely solely on individual behavioral change strategies. Effective prevention will also require policies that address broader socioeconomic and environmental conditions, particularly in rapidly urbanizing settings where structural factors increasingly shape diabetes risk.

The positive association we found between wealth and T2DM risk indicates that economic development alone will not solve the diabetes epidemic in LMICs. Rather, as suggested by Popkin et al. [29], policies must focus on guiding the nutrition transition by implementing targeted health education and promotion initiatives for affluent populations who are currently at higher risk. Specifically, diabetes prevention programs in LMICs should be designed to reach wealthier individuals through workplace interventions, private healthcare settings, and media channels frequented by higher socioeconomic groups. This approach aligns with recommendations by Manne‐Goehler et al. [39], who emphasized the need for diabetes prevention efforts that target the socioeconomic groups most affected within each country's specific development context.

The substantial contextual effects we observed, with 32.2% of diabetes risk variation attributable to neighborhood factors, highlight the critical importance of place‐based interventions. Urban planning and development policies must be reoriented to create health‐promoting environments, especially in rapidly urbanizing areas. As Swinburn et al. [40] argue in their systems approach to obesity prevention, “changing the food and physical activity environments” is necessary to address the structural drivers of metabolic diseases. This could include implementing zoning regulations to limit fast‐food outlet density in residential areas, ensuring adequate green spaces for physical activity, and improving access to affordable healthy foods through targeted subsidies and urban agriculture initiatives.

For neighborhoods with high socioeconomic disadvantage, which our study identified as having significantly higher T2DM risk, targeted community‐level interventions are needed. The WHO's “Urban HEART” (Health Equity Assessment and Response Tool) [41] provides a framework for identifying and addressing neighborhood‐level health inequities. Applying this approach to diabetes prevention could involve conducting community health needs assessments, establishing community health worker programs focused on diabetes screening and management, and developing community‐based lifestyle intervention programs tailored to local contexts.

The substantial urban–rural disparities in diabetes risk we observed call for urban health policies that specifically address the metabolic health challenges of rapid urbanization. Miranda et al. [42] propose a healthy urbanization framework that includes policies to ensure adequate infrastructure for physical activity, regulations on food marketing and availability, and urban design principles that promote active transport. Implementation of such policies could help mitigate the urban health penalty that currently contributes to elevated diabetes risk in urban areas of LMICs.

The interplay between individual and contextual socioeconomic factors we identified suggests the need for health system responses that span the socioeconomic spectrum but are tailored to different risk profiles. Previous studies have argued that health systems in LMICs must simultaneously address the double burden of undernutrition and overnutrition, often within the same communities [43]. This requires integrated approaches that combine traditional infectious disease control with emerging noncommunicable disease prevention.

Our findings support the value of socioeconomically stratified diabetes screening and prevention programs. Given the higher diabetes risk we observed among wealthier groups, opportunistic screening in formal employment settings and private healthcare facilities could effectively reach high‐risk populations. Concurrently, community‐based screening initiatives in disadvantaged neighborhoods are needed to address the contextual risk factors we identified. The WHO's Package of Essential Noncommunicable Disease Interventions (PEN) [44] provides a framework for implementing such stratified approaches in resource‐constrained settings.

To establish causal relationships between socioeconomic exposures and T2DM risk, longitudinal studies are urgently needed in LMICs. Cross‐sectional analyses like ours identify important associations but cannot determine temporal sequences or account for potential reverse causality. As Ebrahim et al. [45] argue in their review of noncommunicable disease research in LMICs, high‐quality cohort studies are essential for understanding disease etiology and developing evidence‐based interventions. Future research should establish prospective cohorts that can track how changes in individual and contextual socioeconomic factors influence diabetes incidence over time.

Specifically, longitudinal studies should investigate how economic development trajectories at the country level interact with individual socioeconomic mobility to shape diabetes risk. This approach could help identify critical windows for intervention during socioeconomic transitions. The PURE study [46] provides a valuable model for such research, having already demonstrated how associations between socioeconomic factors and cardiovascular outcomes vary by country income level.

### 4.4 Study Strengths and Limitations

Our study offers several noteworthy methodological and substantive strengths that enhance the validity and relevance of its findings. First, the use of nationally representative data from 14 LMICs across diverse geographic regions significantly bolsters the external validity of our results. This multicountry approach captures a broad spectrum of socioeconomic and cultural contexts, enabling more generalizable conclusions about diabetes determinants in developing settings than would be possible from single‐country studies. As Basu et al. [47] note in their global diabetes projection study, incorporating data from multiple countries with varying levels of economic development provides crucial insights into the heterogeneous patterns of diabetes emergence across diverse settings.

The large sample size (*N* = 1,090,328 individuals from 38,174 neighborhoods) provides substantial statistical power for detecting even modest associations and enables precise estimation of effect sizes. This robust sample allows for confident interpretation of both significant and nonsignificant findings. Szklo [48] emphasize that large sample sizes are particularly important for multilevel analyses, where statistical power depends on both the number of individuals and the number of higher‐level units (neighborhoods in our case).

The multilevel analytical approach represents a significant methodological strength, allowing simultaneous examination of individual and contextual effects while accounting for the clustering of individuals within neighborhoods. This approach addresses what Subramanian et al. [49] identify as the atomistic fallacy (ignoring group‐level effects) and the ecological fallacy (inferring individual‐level relationships from aggregate data). By employing multilevel models, we can disentangle the independent contributions of individual SEP and neighborhood context to diabetes risk, providing a more comprehensive understanding of how these factors interact across levels.

Our study benefits from the standardized measurement procedures employed in the DHS, ensuring comparability across countries. The DHS program's rigorous methodology, including standardized questionnaires, biomarker collection protocols, and quality control procedures, enhances the reliability and validity of our measures. Corsi et al. [50] highlight the methodological strengths of the DHS program in their comprehensive profile, noting its consistent approach to sampling, data collection, and processing across diverse settings.

Finally, our meta‐analyses complement the multilevel models by providing country‐specific estimates and quantifying between‐country heterogeneity in socioeconomic associations. This approach aligns with recommendations by Thompson [51] for transparency in reporting cross‐national comparisons, enabling readers to assess both the overall patterns and the country‐level variations in socioeconomic determinants of diabetes.

Despite these strengths, several limitations warrant consideration when interpreting our findings. First, the cross‐sectional design precludes causal inference regarding the relationships between socioeconomic factors and diabetes. Temporal ambiguity, particularly for individual‐level socioeconomic indicators, raises the possibility of reverse causality; diabetes may influence SEP through employment limitations, healthcare costs, and reduced earning capacity. As Kawachi et al. [52] note in their review of social epidemiological methods, establishing the temporal sequence of socioeconomic exposures and health outcomes requires longitudinal data that can track changes in both domains over time.

FBG measurements were only available for a subset of participants, with diabetes classification partly based on self‐reported diagnosis. This may introduce potential misclassification bias, particularly if access to diagnosis varies by socioeconomic status, potentially influencing the observed associations. Undiagnosed diabetes remains a significant concern in LMICs. Beagley et al. [53] estimate that globally, nearly half of all diabetes cases remain undiagnosed, with even higher proportions in low‐resource settings. This underdiagnosis may vary systematically by SEP, potentially attenuating or amplifying the observed socioeconomic gradients. Specifically, wealthier individuals may have greater access to diagnostic services, leading to higher reported prevalence even if true biological prevalence is similar across socioeconomic groups. In addition, the DHSs primarily include individuals aged 15–49 years, which may limit the generalizability of the findings to older populations where the prevalence of T2DM is typically higher. Furthermore, the pooled analysis combines surveys conducted over a 10‐year period (2013–2023), which may introduce temporal heterogeneity and limit the direct comparability of prevalence estimates across countries.

Our measurement of SEP at the individual level relied primarily on asset‐based wealth indices rather than income or expenditure data. While wealth indices are widely used in LMIC research and have been validated by Filmer and Pritchett [54], they may not fully capture the dynamic aspects of economic resources that influence diabetes risk. As Howe et al. [55] argue in their methodological review, different dimensions of SEP may have distinct relationships with health outcomes, and asset‐based measures may not adequately reflect current consumption patterns relevant to diabetes risk.

Additionally, BMI was classified using standard WHO cut‐offs (overweight ≥25.0 kg/m^2^, obese ≥30.0 kg/m^2^) uniformly across all countries. However, Asian‐specific thresholds (overweight ≥23.0 kg/m^2^) are recommended for populations in India, Bangladesh, and the Philippines, where cardiometabolic risk arises at lower BMI values than in European populations. Applying WHO standard cut‐offs in these countries may underclassify overweight and obesity, potentially attenuating the observed associations between BMI and T2DM risk in South and East Asian samples. This approach was necessary to ensure cross‐national comparability, but readers should interpret BMI‐related findings for these countries with this caveat in mind.

At the neighborhood level, our delineation of units relied on administrative boundaries (primary sampling units) defined in the DHS, which may not correspond to meaningful socioeconomic or cultural communities. This limitation reflects what Kwan [56] terms the uncertain geographic context problem, the challenge of defining contextual units that accurately capture the environmental exposures relevant to the health outcome under study. Administrative boundaries may obscure important within‐neighborhood heterogeneity and cross‐boundary influences that shape diabetes risk.

Our measure of neighborhood socioeconomic disadvantage, while comprehensive in incorporating education, employment, and wealth dimensions, may not capture all relevant aspects of neighborhood environments that influence diabetes risk. Food environments, recreational facilities, healthcare access, and built environment characteristics were not directly measured. As suggested by Diez Roux and Mair [57], these unmeasured neighborhood features may mediate or confound the observed associations between neighborhood SEP and diabetes.

While our study included 14 countries, this sample represents only a fraction of all LMICs, potentially limiting generalizability to regions underrepresented in our data, particularly Latin America and the Caribbean (represented by Haiti alone). Furthermore, the included countries span a range of development levels within the LMIC category, potentially obscuring nonlinear relationships between development stage and diabetes socioeconomic patterning. As Ezzati et al. [25] demonstrate in their analysis of obesity trends, the socioeconomic gradient of noncommunicable disease risk factors may reverse at different stages of economic development, a nuance our cross‐sectional multicountry approach may not fully capture.

Finally, our study lacks direct measures of potential mediating mechanisms linking SEP to diabetes risk, such as dietary patterns, physical activity, psychosocial stress, and healthcare access. This limitation constrains our ability to elucidate the pathways through which socioeconomic factors influence diabetes outcomes. As Stringhini et al. [58] demonstrate in their analysis of health behavior mediation in socioeconomic inequalities, understanding these pathways is crucial for designing effective interventions that target the appropriate mediating factors.

Despite these limitations, our study provides valuable insights into the multilevel socioeconomic determinants of T2DM in LMICs. The large, diverse sample and robust analytical approach support the validity of our core findings while suggesting important directions for future research that can address these methodological challenges.

## 5 Conclusion

This study provides evidence that both individual and contextual socioeconomic factors independently influence T2D in LMICs. The substantial neighborhood effects we identified, accounting for nearly one‐third of the total variation in diabetes risk, highlight the critical importance of place in shaping health outcomes in these settings. Our findings challenge simplistic assumptions about socioeconomic gradients in health, demonstrating that in transitioning economies, higher wealth may initially confer greater diabetes risk before the typical protective effects emerge.

The complex interplay between individual characteristics and neighborhood environments underscores the need for multilevel interventions that simultaneously address behavioral risk factors and contextual determinants. Urban areas and disadvantaged neighborhoods warrant particular attention, as these contexts independently increase diabetes risk beyond individual factors. Public health strategies must evolve beyond individual‐focused behavioral interventions to incorporate policies addressing the socioeconomic and built environments in which health behaviors develop.

As LMICs continue to experience rapid socioeconomic and epidemiological transitions, understanding the shifting patterns of diabetes risk across socioeconomic strata becomes increasingly important. Future longitudinal research is needed to establish causal relationships and identify critical intervention points during these transitions. Ultimately, effective diabetes prevention in LMICs will require coordinated efforts across sectors to create health‐promoting environments while addressing the complex socioeconomic determinants that drive this growing epidemic.

## Author Contributions

R.F. and O.A.U. conceived the idea. R.F. and O.A.U. acquired, processed, managed, and prepared data for further analysis. O.A.U. developed and designed the methodology. R.F. performed all analyses with support from O.A.U. R.F. investigated and interpreted the results. A.A., M.H., and T.M.B. provided support and feedback throughout the work. R.F. wrote the first version of the manuscript. R.F., O.A.U., A.A., M.H., and T.M.B. provided feedback, substantively revised the manuscript, and contributed to the final version of the work.

## Conflicts of Interest

The authors declare that the research was conducted without any commercial or financial relationships that could be considered potential conflicts of interest.

## Data Availability

The Demographic and Health Surveys, which are nationally representative household surveys carried out in low‐ and middle‐income nations, provided the data for this cross‐sectional analysis, available upon application (https://www.dhsprogram.com/data/Getting‐Started.cfm).
